# Investigating of the Phytoconstituents, Anti‐*α*‐Glucosidase, Antilipase, Anti‐*α*‐Amylase, and DPPH Radical Scavenging Activities of Extracts From *Eriobotrya japonica*

**DOI:** 10.1155/bmri/8888528

**Published:** 2025-08-31

**Authors:** Nidal Jaradat, Mustafa Ghanim, Mohammed Hawash, Johnny Amer, Malik Alqub, Belal Rahhal, Majdi Dwikat, Fatimah Hussein, Linda Issa, Maha Rabayaa, Mohammad Abuawad, Mohammad Halayqa, Wad Zaghloul, Esraa Hamdan

**Affiliations:** ^1^ Faculty of Pharmacy, An-Najah National University, Nablus, State of Palestine, najah.edu; ^2^ Faculty of Medicine and Allied Medical Sciences, An-Najah National University, Nablus, State of Palestine, najah.edu

**Keywords:** *α*-Amylase, *α*-Glucosidase, *Eriobotrya japonica*, free radicals, phenols, phytochemicals, tannins

## Abstract

Recent research reported inhibitory effects of *Eriobotrya japonica* leaf extracts on DPPH free radicals and *α*‐amylase, *α*‐glucosidase, and lipase enzymes. These enzymes were linked to the etiology of diabetes mellitus, obesity, and oxidative stress. The current study is aimed at determining the phytocontents of *E. japonica* aqueous extract leaves and exploring their potential antioxidant, anti‐*α*‐amylase, anti‐*α*‐glucosidase, and antilipase activity using reference phytochemical and biochemical assays. The phytochemical tests on the *E. japonica* aqueous extract confirmed the presence of alkaloids, glycosides, tannins, saponins, phytosteroids, carbohydrates, and phenols. The total phenol and tannin contents of the *E. japonica* aqueous extract were 21.64 ± 0.89 mg of GAE/g and 1.72 ± 1 mg of CAE/g, respectively. *E. japonica* aqueous extract had inhibitory effects on DPPH free radicals (IC_50_ of 7.7 ± 3.11 *μ*g/mL) and *α*‐amylase (IC_50_ = 141 ± 0.35 *μ*g/mL) compared with Trolox and acarbose (6.3 ± 0.12 and 28.18 ± 1.22 *μ*g/mL, respectively). Moreover, *E. japonica* aqueous extract inhibited *α*‐glucosidase (IC_50_ = 39.81 ± 0.74 vs. 37.15 ± 0.33 *μ*g/mL inhibition by acarbose) and lipase (IC_50_ = 316.2 ± 0.87 vs. 12.3 ± 0.33 *μ*g/mL inhibition by orlistat). In conclusion, the current results suggest that *E. japonica* aqueous extract possesses strong antioxidant, anti‐*α*‐glucosidase, anti‐*α*‐amylase, and antilipase activities with potential applications in the treatment and prevention of obesity, diabetes mellitus, and oxidative stress.

## 1. Introduction

*Eriobotrya japonica* (Thunb.) Lindl belongs to the Rosaceae family, which has 91 genera and roughly 4828 species worldwide [1]. *E. japonica* is a perennial evergreen tree with leathery, simple, sharp, and veiny leaves. The leaf’s top is smooth, but the bottom is downy and covered in small hairs. It originated in southeastern China and has since become naturalized in Korea, Japan, India, and many other nations [2]. Its cultivation, however, has spread practically everywhere in the world, and it can be found in countries like Spain, India, Egypt, Cyprus, Italy, Australia, Mexico, and Tunisia [3].

As a medicinal herb, *E. japonica* serves its purpose. From ancient times, the leaves of this plant have been utilized in traditional Chinese medicine [4] as a remedy for respiratory diseases, as well as gastrointestinal, cardiovascular, and metabolic disorders [5–7]. Several biologically active compounds have been reported in the leaves of this plant, including triterpenes, sesquiterpenes, flavonoids, tannins, and megastigmane glycosides. The leaves of *E. japonica* are reported to have anti‐inflammatory, hypoglycemic, antioxidant, antitumor, and antiviral activities [8–10]. Pande and Akoh’s study [11] reported that the *E. japonica* leaf extract contains flavonoids (epicatechin, catechin, and quercetin) and phenolic acids, namely, *p*‐coumaric, caffeic, ellagic, and gallic acids [4].

In diabetes, one of the goals of reducing hyperglycemia is to reduce the activity of *α*‐glucosidase, which is responsible for the hydrolysis of carbohydrates. *α*‐Glucosidase inhibitors delay the intestinal absorption of glucose, thereby limiting the fluctuation of postprandial blood glucose [12]; moreover, antidiabetic activity can be assessed by *α*‐amylase inhibitory effect [7].

Oxidative stress, resulting from an imbalance between the production of reactive oxygen species (ROS) and the body’s ability to neutralize them, is implicated in the pathophysiology of obesity, diabetes, and cancer [13]. By producing adipokines and pro‐inflammatory cytokines, extra adipose tissue in obesity might exacerbate oxidative stress. Insulin resistance and the onset of Type 2 diabetes may be exacerbated by this oxidative damage [14]. A popular technique for evaluating a substance’s antioxidant qualities is the 2,2‐diphenyl‐1‐picrylhydrazyl (DPPH) free radical scavenging test, which measures a substance’s ability to donate hydrogen or scavenge free radicals. It is widely used to assess the antioxidant activity of different compounds and is recognized for being quick, easy, and cost‐efficient [15].

The lipolytic pancreatic lipase enzyme is synthesized and secreted by the pancreas, plays a key role in the efficient digestion of lipids, and is responsible for the hydrolysis of 50%–70% of total dietary lipids. The antilipase effect is one of the most widely studied mechanisms in determining the potential efficacy of natural products as antiobesity agents [16].

In most cases, extracts of medicinal plants demonstrate therapeutic action due to the combined effects of their many components rather than a single active ingredient. Many investigations have shown that the medicinal plants’ constituents are affected by various factors, including the part of the plant used, extraction methods, geographical location, soil type, rainfall, and other environmental factors [17–20].

Therefore, the current work is aimed at investigating for the first time the *α*‐glucosidase, lipase, *α*‐amylase, and DPPH free radical inhibitory activities as well as the phytoconstituents of *E. japonica* aqueous extract collected from Palestine.

## 2. Material and Methods

### 2.1. Sample and Extraction Procedure

The *E. japonica* leaves were harvested from the Hebron region of Palestine during the plant’s flowering time in May 2021. Pharmacognosist Dr. Nidal Jaradat identified and confirmed this plant in the Herbal Products Laboratory of the Department of Pharmacy at An‐Najah National University (voucher number: Pharm‐PCT‐2785), and the plant sample was deposited in the same laboratory. The freshly collected leaves were cleaned with tap water and then dried for 3 weeks, in the shade at a controlled humidity (55 ± 5 RH) and temperature (25 ± 2^°^C). The dried leaves were powdered using a mechanical blender (Molineux model, Uno, China). The plant was extracted with distilled water, where 400 g of dried *E. japonica* leaves was weighed and steeped in 4000 mL of distilled water for 72 h. The extract was filtered twice using filter papers (Whitman No. 1, United States). The obtained aqueous extract was frozen in the refrigerator for 3 days and then dried in a freeze‐drier (Millrock Technology BT85, China) for 4 days. Then, the dried extract was preserved in a closed container at 4°C.

Finally, using this formula, the extract yield was determined:

Yield %=weight of plant extractweight of dry plant×100%

The *E. japonica* extract yield was 11.17%.

### 2.2. Chemicals and Reagents

Porcine pancreatic *α*‐amylase enzyme, dimethyl sulfoxide (DMSO), Trolox ([s]‐[‐]‐6‐hydroxy‐2,5,7,8‐tetramethylchroman‐2‐carboxylic acid), Folin–Ciocalteu’s reagent, porcine pancreatic lipase enzyme, dinitrosalicylic acid (DNSA), and DPPH were purchased from Sigma‐Aldrich (Germany). Millon’s and Benedict’s reagents were purchased from Gadot (United States). Gallic acid, methanol, Dragendorff reagent, and sodium carbonate were brought from Merck (Darmstadt, Germany). Ninhydrin solution, Molish’s reagent, H_2_SO_4_, chloroform, magnesium ribbon, NaOH, FeCl_3_, HCl, and iodine solution were obtained from Alfa Aesar (England). The purity of all purchased chemicals and reagents was higher than 99.0%.

### 2.3. Preliminary Phytochemical Assessment

The aqueous extract of *E. japonica* leaves was screened using reference analytical tests for the availability of the main natural phytochemical classes including alkaloids, glycosides, tannins, saponins, phytosteroids, terpenoids, phenols, flavonoids, protein, and amino acids [21, 22].

### 2.4. Quantitative Total Phenol Content

The total phenolic content of *E. japonica* aqueous extract was evaluated calorimetrically using the Folin–Ciocalteu procedure method, in which gallic acid was used as a reference molecule [23]. For this analysis, a concentration of 1 mg/mL aqueous solution was prepared from the plant’s aqueous extract. The reaction mixtures were prepared by mixing 0.5 mL of the prepared aqueous solution, 2.5 mL of 10% Folin–Ciocalteu testing agent dissolved in water, and 2.5 mL of 7.5% NaHCO_3_ aqueous solution. Then, the mixtures were left at a constant temperature of 45°C for 45 min. The absorbance at a wavelength of 765 nm was then determined by spectrophotometry. The absorbance values were reported as the mean values calculated from triple tests. The absorbance values of the standard molecule, gallic acid, were determined using the same method, and then the required calibration curve was constructed. Based on the measured absorbance, the concentration of the gallic acid equivalent was calculated in milligrams of gallic acid equivalents per gram for the studied plant sample.

### 2.5. Determination of Total Tannin Content

The condensed tannin content of *E. japonica* aqueous extract was estimated based on Sun et al.’s [24] method. In brief, 0.5 mL of 10, 30, 50, 70, and 100 *μ*g/mL concentrations of plant extract was added to 3 mL of 4% vanillin–methanol solution and 1.5 mL of concentrated HCl. Following a 15‐min standing period, absorption was measured at 500 nm wavelength against a methanol‐containing blank. The plant sample was examined in triplicate, and the mean tannin content was estimated concerning catechin equivalents (milligrams of catechin acid equivalents per gram of plant extract).

### 2.6. DPPH Inhibitory Assay

The *E. japonica* extract’s DPPH free radical scavenging capacity was evaluated using the DPPH inhibition assay. The *E. japonica* extract solution was gradually diluted in methanol as a solvent to maintain concentrations of 0, 5, 7, 10, 30, 50, 80, and 100 *μ*g/mL. A total of 1 mL of each concentration was prepared. Then, 1 mL of methanol and 1 mL of 0.002% methanolic DPPH solution were added to each prepared concentration, resulting in 3 mL as the final volume. After a 30‐min incubation period in the dark, the absorbance at a 517 nm wavelength was spectrophotometrically determined. The following equation was used to calculate the inhibition percentage:

%DPPH inhibition=AB−AE×100%AB

where AB is the recorded absorbance of the blank solution and AE is the recorded absorbance of the *E. japonica* sample solution.

The plant extract’s antioxidant half‐maximal inhibitory concentration (IC_50_) was calculated utilizing BioDataFit Edition 1.02 [25].

### 2.7. *α*‐Amylase Inhibitory Assay

This assay was performed according to the method modified by McCue and Shetty [26]. The following concentrations were utilized in this study: 10, 50, 70, 100, and 500 *μ*g/mL. These concentrations were prepared from a 1000 *μ*g/mL solution that was previously produced by dissolving plant extract in a few milliliters of 10% DMSO, and then another part of the extract was dissolved in a buffer (0.02 M of Na_2_HPO_4_/NaH_2_PO_4_, 0.006 M NaCl, at pH 6.9). After that, 2 units/mL of porcine pancreatic *α*‐amylase enzyme solution was prepared freshly in 10% DMSO. A working solution was prepared by mixing 0.2 mL of enzyme solution with 0.2 mL of the hydrophilic extract and then incubating for 10 min at 30°C. Following the incubation, 0.2 mL of a freshly prepared 1% (w/v) aqueous starch solution was added to each working solution. After that, the final solution was incubated for at least 3 min, and 0.2 mL of DNSA was added to stop the reaction. Each working solution was then diluted with 5 mL of distilled water and boiled for 10 min in a water bath at 90°C. After cooling the mixture to room temperature, the absorbance at 540 nm was measured. Similar steps were used to prepare the blank, but 0.2 mL of the previously mentioned buffer replaced the plant extract volume. Acarbose was used as the standard reference.

The *α*‐amylase inhibitory activity was calculated using the following equation:

%α‐Amylase inhibitory activity=AB−AE×100%AB

where AB is the absorbance of the blank and AE is the absorbance of *E. japonica* aqueous extract.

### 2.8. Porcine Pancreatic Lipase Inhibition Assay

The antilipase assay was conducted according to Bustanji et al.’s studies with a few modifications [27]. A stock solution was prepared by diluting 1 mg/mL of *E. japonica* aqueous extract with 10% DMSO. From this stock solution, concentrations of 50,100, 200, 300, and 400 *μ*g/mL were prepared. In addition, a 1 mg/mL concentrated solution of pancreatic lipase was combined with a Tris‐HCl buffer solution. A solution containing 20.9 mg of *p*‐nitrophenyl butyrate was prepared by mixing it with 2 mL of acetonitrile. Next, 0.1 mL of porcine pancreatic lipase enzyme with a concentration of 1 mg/mL was combined with 0.2 mL of the plant fraction. The resulting mixture was subsequently diluted to a volume of 1 mL by adding a Tris‐HCl solution and maintained at a temperature of 37°C for 15 min. Subsequently, a volume of 0.1 mL of *p*‐nitrophenyl butyrate was introduced into each working sample. The mixtures were left to incubate for 30 min at 37°C. The activity of pancreatic lipase was assessed by quantifying the breakdown of *p*‐nitrophenolate to *p*‐nitrophenol at a wavelength of 405 nm, using a UV/visible spectrophotometer. The identical procedure was carried out utilizing orlistat, which was employed as a positive control. Furthermore, each of the examined samples underwent analysis three times.

### 2.9. *α*‐Glucosidase Inhibitory Activity

A stock solution was prepared by dissolving 1 mg of the *E. japonica* aqueous extract in 1 mL of phosphate buffer. The solution obtained was diluted with phosphate buffer to obtain various concentrations (100, 200, 300, 400, and 500 *μ*g/mL). A total of 20 *μ*L of the stock solution and *α*‐glucosidase solution (with a concentration of 1 unit/mL) was combined with 50 *μ*L of phosphate buffer. The mixture was then placed in a water bath at a temperature of 37°C and incubated for 15 min. Subsequently, a volume of 20 *μ*L of P‐NPG solution was introduced into the mixture and allowed to react for 20 min at a temperature of 37°C. The reaction was then stopped by adding 50 *μ*L of a 0.1 M Na_2_CO_3_ solution. The empty solution was prepared by substituting the plant extract solution with phosphate buffer. Acarbose was used as a reference substance, and the absorbance for the samples was measured at a wavelength of 405 nm using a UV‐Vis spectrophotometer. The *α*‐glucosidase inhibitory activity was determined by applying the following formula:

I %=ABSblank–ABStest ABSblank∗100%

where *I* (*%*) is the percentage inhibition of *α*‐glucosidase [28].

### 2.10. Statistical Analysis

The quantitative phytochemical and biological assessments of the aqueous extract from *E. japonica* were iteratively conducted in triplicate. The mean values of the obtained results, accompanied by their respective standard deviations (±SD), were subsequently computed using Student’s *t*‐test. A *p* value of < 0.05 was considered significant.

## 3. Results

### 3.1. Phytochemical Screening

The preliminary phytochemical tests on the *E. japonica* aqueous extract showed that alkaloids, glycosides, tannins, saponins, phytosteroids, carbohydrates, and phenols were identified in the extract, as shown in Table 1.

**Table 1:** Preliminary phytochemical screening of *E. japonica* aqueous extract.

**Phytochemical classes**	**Results**
Alkaloids	+
Glycosides	+
Tannins	+
Saponin	+
Phytosteroids	+
Terpenoids	−
Protein and amino acid	−
Phenol	+
Flavonoid	−

*Note:* (+) = present and (−) = absent.

### 3.2. Quantitative Analysis

A calibration curve of gallic acid was constructed based on absorption values of several concentrations of standard gallic acid.

In addition, a calibration curve of catechin was constructed based on the absorption values of several concentrations of the standard catechin.

The total phenol and tannin contents (gallic acid equivalent per gram GAE) of the *E. japonica* aqueous extract are 21.64 ± 0.89 mg of GAE/g and 1.72 ± 1 mg of CAE/g, respectively.

### 3.3. DPPH Free Radical Inhibitory Effect

**Figure 1:**
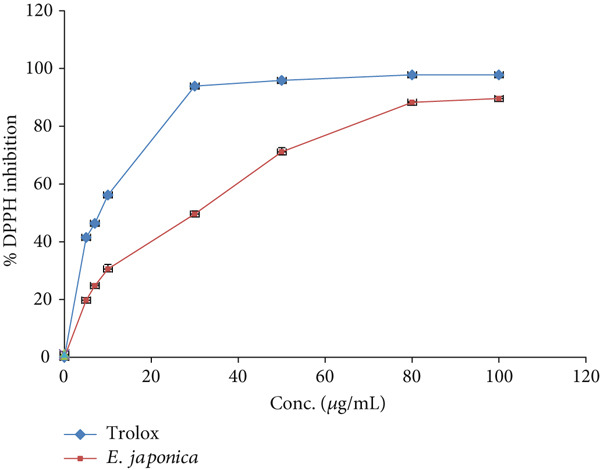
DPPH inhibition effect of *E. japonica* hydrophilic extract and Trolox.

At the 100 *μ*g/mL concentration, *E. japonica* hydrophilic extract inhibited the activity of DPPH free radicals by 89.60 ± 0.8*%* compared with Trolox, which inhibited DPPH free radicals by 97.83 ± 0.02*%* (Figure 1). The obtained IC_50_ values of *E. japonica* extract and Trolox were 17.7 ± 3.11 and 6.3 ± 0.12 *μ*g/mL, respectively.

### 3.4. *α*‐Amylase Suppressant Effect

**Figure 2:**
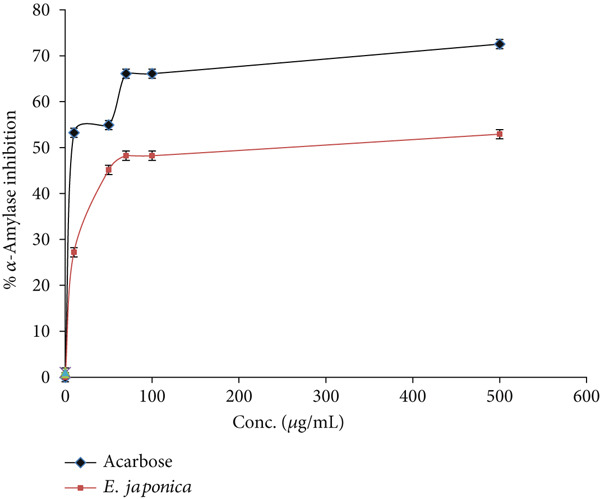
*α*‐Amylase inhibitory activity by *E. japonica* hydrophilic extract and acarbose.

A concentration of 500 *μ*g/mL of *E. japonica* aqueous extract inhibited the *α*‐amylase activity by 52.92 ± 0.025*%* compared with acarbose, which at the same concentration inhibited the activity of *α*‐amylase by 72.54 ± 1.37*%* as shown in Figure 2. The IC_50_ inhibitory activity values of *E. japonica* hydrophilic extract and acarbose on the *α*‐amylase were 141 ± 0.35 and 28.18 ± 1.22 *μ*g/mL, respectively.

### 3.5. *α*‐Glucosidase Suppressant Effect

**Figure 3:**
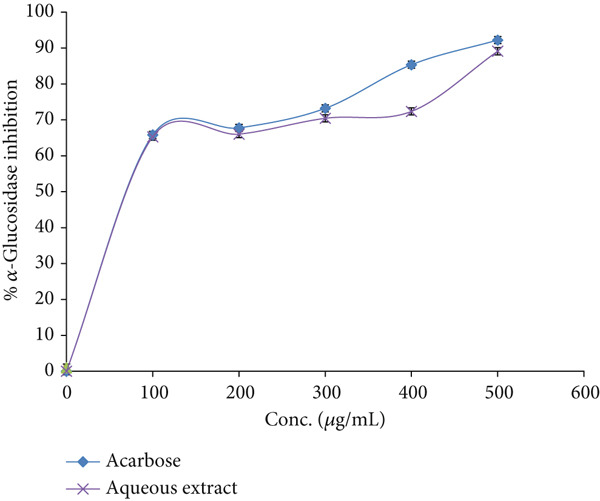
*α*‐Glucosidase inhibitory activity by *E. japonica* hydrophilic extract and acarbose.

At a concentration of 500 *μ*g/mL, the aqueous extract of *E. japonica* demonstrated an 89.11 ± 0.82*%* inhibition of *α*‐glucosidase activity. In contrast, acarbose, at the same concentration, exhibited a slightly higher inhibition of 92.22 ± 0.11*%*, as demonstrated in Figure 3. The IC_50_ values, which represent the concentration causing 50% inhibition, for *E. japonica* aqueous extract and acarbose against *α*‐glucosidase were 39.81 ± 0.74 and 37.15 ± 0.33 *μ*g/mL, respectively.

### 3.6. Lipase Inhibitory Effect

**Figure 4:**
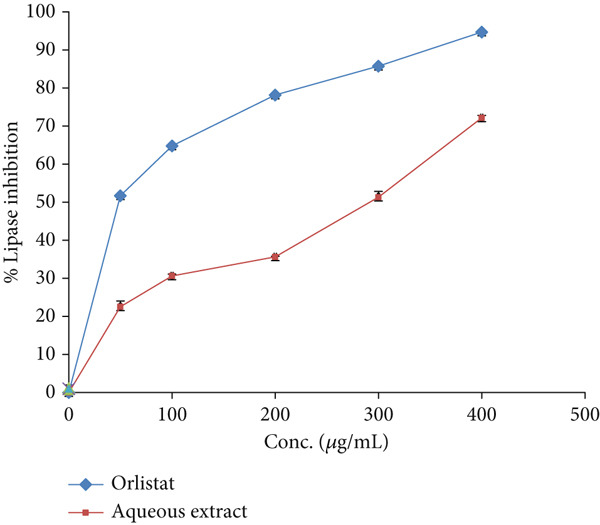
Lipase inhibitory activity by *E. japonica* hydrophilic extract and orlistat.

At a concentration of 400 *μ*g/mL, the aqueous extract of *E. japonica* showed a lipase inhibition of 72.13 ± 0.67*%*. In contrast, orlistat, at the same concentration, exhibited a higher inhibition of 94.67 ± 0.31*%*, as demonstrated in Figure 4. The IC_50_ values, which represent the concentration causing 50% inhibition, were 316.2 ± 0.87 *μ*g/mL for *E. japonica* aqueous extract and 12.3 ± 0.33 *μ*g/mL for orlistat against lipase.

## 4. Discussion

The present study provides evidence of the phytochemical composition and biological activities of *E. japonica* aqueous leaf extract collected from Palestine. The extract was found to be rich in secondary metabolites such as phenols, tannins, glycosides, alkaloids, and saponins, which are known for their therapeutic potential. These findings are consistent with previous studies that reported the presence of phenolic acids (e.g., gallic, caffeic, and *p*‐coumaric acids), flavonoids (such as quercetin, catechin, and epicatechin), and triterpenes in *E. japonica* leaves [8, 11]. The total phenolic content of the current study extract (21.64 ± 0.89 mg GAE/g) was higher than what was reported by Mogole et al. [29] and Pawłowska et al. [30], who found phenolic contents of 6.05 mg GAE/100 g and 16.4 mg GAE/g, respectively, but slightly lower than the values obtained by Hong et al. [28]. This is likely due to differences in extraction techniques, geographical origin, and environmental factors such as UV exposure and soil composition [31, 32]. Tannins, which were also detected, have been recognized for their ability to modulate oxidative stress, inhibit digestive enzymes, and contribute to the management of diabetes and cancer [33, 34].

The antioxidant activity of *E. japonica* aqueous extract, as evidenced by its DPPH radical scavenging activity (IC_50_ = 17.7 ± 3.11 *μ*g/mL), supports the traditional use of this plant in mitigating oxidative stress–related disorders. Although slightly less potent than Trolox (IC_50_ = 6.3 ± 0.12 *μ*g/mL), the current extract showed higher antioxidant capacity compared to previously reported methanolic leaf extracts (IC_50_ ≈ 35.5 *μ*g/mL) [35]. This high activity can be attributed to the abundance of phenolic compounds, which act as free radical scavengers, hydrogen donors, and metal chelators [36, 37]. Previous studies have established a correlation between phenolic content and antioxidant potential, with higher phenolic content often linked to improved radical scavenging abilities [31, 38]. Our findings align with studies suggesting that *E. japonica* seed extracts also possess antioxidant activity, with some reports showing even lower IC_50_ values [38], indicating that different parts of the plant may contribute complementary bioactivities.

In addition to its antioxidant effects, *E. japonica* extract demonstrated noteworthy enzyme inhibitory activities. The *α*‐amylase inhibitory activity observed (IC_50_ = 141 *μ*g/mL) was lower than that of acarbose (IC_50_ = 28.18 *μ*g/mL), but significantly higher than what was previously reported for methanolic extracts (IC_50_ = 453 *μ*g/mL) [37]. This suggests that aqueous extracts may retain bioactive compounds that are particularly effective in targeting *α*‐amylase. As *α*‐amylase plays a critical role in the digestion of carbohydrates and postprandial hyperglycemia [39, 40], natural inhibitors such as those in *E. japonica* could provide a safer alternative to synthetic drugs, which often cause gastrointestinal side effects. Similar enzyme inhibition was reported in plants such as *Tinospora crispa*, *Curcuma longa*, and *Coriandrum sativum* [41, 42], further supporting the potential of *E. japonica* as a functional food or phytotherapeutic agent for diabetes management.

Notably, the *α*‐glucosidase inhibitory activity of our extract (IC_50_ = 39.81 ± 0.74 *μ*g/mL) was nearly equivalent to that of acarbose (IC_50_ = 37.15 ± 0.33 *μ*g/mL). This inhibition is likely due to catechins, theaflavins, and other galloylated compounds identified in *E. japonica* leaves, which was previously recognized as strong *α*‐glucosidase inhibitors [43, 44]. By delaying the hydrolysis of complex carbohydrates and glucose absorption, *α*‐glucosidase inhibitors can effectively reduce postprandial glucose spikes and improve glycemic control [45]. The observed activity of *E. japonica* in the current study is comparable or higher to that of many other medicinal plants traditionally used for diabetes mellitus, which highlights its therapeutic promise.

The lipase inhibitory activity of *E. japonica* (IC_50_ = 316.2 *μ*g/mL) was less than orlistat (IC_50_ = 12.3 *μ*g/mL), but nonetheless significant. This finding is consistent with studies showing that *E. japonica* extracts or fermented tea formulations can suppress lipid absorption, reduce triglyceride levels, and decrease fat accumulation in animal models [44, 46]. For example, Shih et al. [47] reported that *E. japonica* administration in high‐fat‐fed mice improved hyperlipidemia and reversed insulin resistance, while Sharma et al. [46] demonstrated synergistic antiobesity effects when *E. japonica* was combined with *Nelumbo nucifera*. The bioactivity is thought to be mediated by downregulation of lipogenic enzymes and adipokines, as well as improved lipid metabolism [47].

Our findings are supported by Khouya et al. [48], who identified naringenin, procyanidin C1, epicatechin, and rutin as major phenolic compounds in *E. japonica* aqueous extracts and demonstrated its ability to ameliorate hyperglycemia, oxidative stress, and hyperlipidemia in diet‐induced models. Collectively, these results suggest that *E. japonica* possesses multifunctional bioactivities that could be leveraged for the development of nutraceuticals targeting metabolic disorders. However, while our in vitro results are promising, they may not fully reflect the in vivo complexity of absorption, metabolism, and bioavailability. Therefore, further studies, including animal models and clinical trials, are required to confirm the therapeutic potential of *E. japonica*, to isolate and characterize the active compounds, and to investigate their possible synergistic or antagonistic interactions. Also, the current study was conducted using the aqueous extract of the leaves; thus, additional studies should be carried out on nonaqueous extract and, more specifically, on the purified bioactive compounds to ascertain the desired pharmacological effects.

## 5. Conclusion

The current study showed that *E. japonica* hydrophilic extract is rich in secondary metabolic products such as phenols and tannins. It is concluded that the antiamylase and antioxidant activities of the *E. japonica* hydrophilic extract were due to the effect of polyphenols. Therefore, *E. japonica* hydrophilic extract could be considered a potential treatment for diabetes mellitus and oxidative stress–related diseases. Further investigation is recommended to isolate the biologically active chemicals in the *E. japonica* extract to assess their possible toxicological and pharmacological effects in vivo.

## Ethics Statement

We confirm that all procedures utilized in this investigation, including the use of the *E. japonica* plant, were conducted in accordance with the relevant institutional, national, and international guidelines and legislation. The plant was identified and authenticated at An‐Najah National University. A voucher number (Pharm‐PCT‐2785) was deposited in the Pharmacy Department of An‐Najah National University.

## Disclosure

All authors have read and agreed to the published version of the manuscript. All authors have given their consent for publication.

## Conflicts of Interest

The authors declare no conflicts of interest.

## Author Contributions

Conceptualization: N.J.; methodology: N.J. and M.G.; software: M.H. and F.H.; validation: L.I., N.J., and M.G.; formal analysis: N.J., M.G., M.AL., and J.A.; investigation: all authors; resources: N.J.; data curation: N.J., M.H., and M.G.; writing—original draft preparation: N.J., J.A., M.D., M.AB., and M.R.; writing—review and editing: N.J., M.G., M.D., B.R., M.AB., and M.R.; visualization: N.J.; supervision: N.J.; project administration; N.J. and M.G.

## Funding

No funding was received for this manuscript.

## Data Availability

Data collected or analyzed in this investigation are included in this manuscript.
